# Inflammatory Markers Are Positively Associated with Serum* trans*-Fatty Acids in an Adult American Population

**DOI:** 10.1155/2017/3848201

**Published:** 2017-07-11

**Authors:** Mohsen Mazidi, Hong-kai Gao, Andre Pascal Kengne

**Affiliations:** ^1^Key State Laboratory of Molecular Developmental Biology, Institute of Genetics and Developmental Biology, Chinese Academy of Sciences, Chaoyang, Beijing, China; ^2^Institute of Genetics and Developmental Biology, International College, University of Chinese Academy of Sciences (IC-UCAS), West Beichen Road, Chaoyang, China; ^3^Department of General Surgery, The General Hospital of Chinese People's Armed Police Forces, Beijing, China; ^4^Non-Communicable Disease Research Unit, South African Medical Research Council and University of Cape Town, Cape Town, South Africa

## Abstract

**Background and Aim:**

The relationship between serum* trans*-fatty acids (TFAs) and systemic inflammation markers is unclear. We investigated the association of serum TFAs with high sensitivity C-reactive protein (hs-CRP) and fibrinogen in adult Americans.

**Methods:**

The 1999 to 2000 National Health and Nutrition Examination Survey (NHANES) participants with measured data on hs-CRP and fibrinogen were included. TFAs were measured via capillary gas chromatography and mass spectrometry using negative chemical ionization. Analysis of covariance and multivariable-adjusted linear regression models were used to investigate the associations between these parameters, accounting for the survey design.

**Results:**

Of the 5446 eligible participants, 46.8% (*n* = 2550) were men. The mean age was 47.1 years overall: 47.8 years in men and 46.5 years in women (*p* = 0.085). After adjustment for age and sex, mean serum TFAs rose with the increasing quarters of hs-CRP and fibrinogen (both *p* < 0.001). In linear regression models adjusted for age, sex, race, education, marital status, body mass index, and smoking, serum TFAs were an independent predictor of plasma hs-CRP and fibrinogen levels.

**Conclusion:**

A high level of TFAs appears to be a contributor to an unfavourable inflammatory profile. Because serum TFAs concentrations are affected by dietary TFA intake, these data suggest a possible contribution of TFAs intake modulation in the prevention of inflammation-related chronic diseases.

## 1. Introduction

Cardiovascular disease (CVD) and diabetes mellitus (DM) are typically characterized by elevated levels of plasma inflammatory markers [1]. High sensitivity C-reactive protein (hs-CRP) is an acute-phase protein produced by hepatocytes in response to inflammatory cytokines such as interleukin-6 (IL-6) [2]. Circulating markers of inflammation including hs-CRP, tumor necrosis factor-*α*, and some interleukins (IL-6 and IL-1) have been associated with a high risk of CVD [3]. Furthermore, it has been suggested that plasma hs-CRP may serve as a predictor for both CVDs and DM [4].

*trans*-Fatty acids (TFAs) contain at least one double bond in the* trans* configuration between two consecutive carbon atoms. Because humans cannot produce TFAs, their serum levels of TFAs essentially reflect dietary consumption. TFAs occur naturally in fat from ruminant animal meat, milk, and dairy fat and industrially hardened vegetable oils [5]. Dietary exposure to partially hydrogenated vegetable oils occurs via consumption of margarine and industrially processed foods [6]. An observational study [7] and a short-term randomized trial [8] have shown that the intakes of oleic acid (*trans *18:1), linoleic acid (*trans* 18:2), and* trans* 18:1 accounted for 71% of total TFA intake and were positively associated with an increase in systemic inflammatory markers. In addition, increased levels of* trans*-palmitoleic acid (16:1n-7* trans*) have been associated with lesser risk of type 2 diabetes [9]. In these studies, most TFAs originated from outdoor fried foods (18%), cookies, donuts, or sweet rolls (17%), margarine (10%), beef (9%), and crackers (4%).

Studies have reported a direct correlation between serum TFAs and consumption of TFAs [10, 11]. However, epidemiologic studies are limited by the assessment of dietary intake via food frequency questionnaires, a method prone to measurement error [12]. Furthermore, the translation of quantities of food items consumed into their fatty acid content is very sophisticated. Indeed, existing nutrient databases are imperfect and of questionable accuracy on TFAs content of foods. For instance, an average value might not sufficiently define the TFAs content of a generic food item [13]. It has been suggested that fatty acid content of a given food can vary based on cooking methods and industry supply [14]. On the other hand, serum TFAs level might reflect the body's fatty acid composition, quality of dietary fat, and the type of fat consumed over a long period [15]. Hence, evaluating serum TFAs may provide robust findings on the association and may shed light on mechanisms explaining the deleterious impact of TFAs.

A potential link between inflammation, cardiometabolic risk factors, and serum TFAs has been suggested in both animal and human studies [7]. Recent studies have shown that TFAs may change cellular lipid and glucose metabolism, intracellular signaling pathways, and cytokine secretion [16]. However, the relationship between serum TFAs and serum inflammation parameters is unclear [17]. We therefore investigated the association between inflammatory biomarkers (plasma hs-CRP and fibrinogen) and serum TFAs levels in an adult American population.

## 2. Methods

### 2.1. Population

The current cross-sectional study used data from the 1999-2000 cycles of the US National Health and Nutrition Examination Surveys (NHANES), which are conducted on an ongoing basis by US National Center for Health Statistics (NCHS) [18, 19]. The NCHS Research Ethics Review Board approved the NHANES protocol and consent was obtained from all participants [18, 19]. Details on the demographic, socioeconomic, dietary, and health-related characteristics of participants were collected by trained interviewers, using questionnaires administered during home visits [20]. Physical examination was performed at mobile examination centers, where blood sample was drawn from participant's antecubital vein by a trained phlebotomist. hs-CRP and fibrinogen levels were measured with Latex-enhanced nephelometry (Seattle, USA) and Coagamate XC Plus automated coagulation analyzer (Organon Teknika, Durham, NC), respectively. More detailed information on the NHANES protocol is available elsewhere [21, 22]. Analyses were restricted to participants aged 18 years and older.

### 2.2. Serum* trans*-Fatty Acids

Serum TFAs measurements included total (free and esterified) content of selected TFAs [18, 19]. TFAs measurement proceeded through the following sequences. Serum fatty acids were converted into free fatty acids via acidic and alkaline hydrolysis. Fatty acids were then identified based on their chromatographic retention time and specific mass-to-charge ratio of the ion formed. Retention times were thereafter compared against those from known standards [23]. Quantitation was performed with standard solution using stable isotope-labelled fatty acids as internal standards. The following TFAs were measured and used in the current study:* trans*-9-hexadecenoic acid (palmitelaidic acid, C16:1n-7t),* trans*-9-octadecenoic acid (elaidic acid, C18:1n-9t),* trans*-11-octadecenoic acid (vaccenic acid, C18:1n-7t), and* trans*-9-,* trans*-12-octadecadienoic acid (linolelaidic acid, C18:2n-6t, 9t) [18, 19]. Detailed protocol is available in NHANES manual [24].

### 2.3. Statistical Analysis

We applied the CDC protocol for analyzing the complex NHANES data, accounting for the masked variance and using the proposed weighting methodology [25–27]. We computed age and sex mean of TFAs across quarters of hs-CRP and fibrinogen using analysis of covariance (ANCOVA) with Bonferroni correction. To investigate the association of TFAs with CRP and fibrinogen, we used linear regression models adjusted for sex, race, education, marital status, body mass index, and smoking. Groups were compared using analysis of variance and Chi-square tests. All tests were two-sided and *p* < 0.05 was used to characterize statistically significant findings. Data were analyzed using SPSS complex sample module version 22.0 (IBM Corp., Armonk, NY).

## 3. Results

Of the 5446 eligible participants, 46.8% (*n* = 2550) were men. The mean age was 47.1 years overall: 47.8 years in men and 46.5 years in women (*p* = 0.085). With regard to education, 38.2% (*n* = 1863) of the participants were educated beyond high school and 22.5% (*n* = 1096) had completed high school, while 38.9% (*n* = 1896) were not educated to high school level. White people (non-Hispanic) represented 47.2% (*n* = 2327) of the participants, blacks (non-Hispanic) represented 11.9% (*n* = 1035), and Mexican-Americans represented 28.5% (*n* = 1553). In all, 50.6% (2473) of the participants were married, 9.7% (*n* = 475) were widowed, and 7.7% (*n* = 376) were divorced.

Mean and standard deviation of the serum TFAs was 1.90 ± .48 (umol/L) for* trans*-9-hexadecenoic acid, 3.6 ± 0.69 (umol/L) for* trans*-11-octadecenoic acid, 3.4 ± 0.51 (umol/L) for* trans*-9-octadecenoic acid, and 0.99 ± 0.47 (umol/L) for* trans*-9-,* trans*-12-octadienoic acid for those who had information on TFAs, respectively. Mean body mass index was 28.5 ± 6.7 Kg/m^2^ overall: 27.4 ± 5.3 Kg/m^2^ in men and 28.6 ± 6.9 Kg/m^2^ in women.

Concentrations of serum TFAs increased with increasing quarters of both hs-CRP and fibrinogen (all *p* < 0.001, Table 1). After stratification by gender, there was a significant positive association between CRP and all TFAs in both men and women (all *p* < 0.001); however, for fibrinogen, there was significant positive association with TFAs only in men (all *p* < 0.001). After stratification by ethnicity, only non-Hispanic white people and Mexican-Americans displayed significant positive associations between TFAs and both CRP and fibrinogen (all *p* < 0.001).

In adjusted* (age, sex, race, education, marital status, body mass index, and smoking)* linear regression models, significant positive associations were found between* trans*-9-hexadecenoic acid,* trans*-11-octadecenoic acid, and* trans*-9-octadecenoic acid and serum hs-CRP (*p* < 0.001) and between* trans*-9-hexadecenoic acid and* trans*-11-octadecenoic acid in fibrinogen levels (*p* < 0.001).

## 4. Discussion

The potential adverse impact of TFAs on CVD and DM risk has been known since the early 1990s [28, 29]. There are limited data on the associations between serum TFAs and inflammatory status. In this large population-based study, we have evaluated the association between serum markers of inflammation and serum TFAs. Both hs-CRP and fibrinogen were positively associated with TFAs, even after adjusting for potential confounding factors.

Previous studies have reported that TFAs may induce endothelial dysfunction and that this may be related to an upregulation of proinflammatory molecules, linking vascular inflammation and thrombosis [30–32]. Studies in animals and in vitro studies have reported that TFAs may stimulate inflammatory processes [33], with suggestion that, in TFA-exposed blood vessels, inflammation and oxidative stress may trigger prothrombogenic activity of endothelial cells, which then exceeds the antithrombogenic activity [33]. Dietary fatty acid consumption has been reported to alter platelet aggregation [34]. It has been suggested that TFAs may increase the formation of proinflammatory cytokines through activation of nuclear factor-*κ*B (NF-*κ*B) signaling and induce endothelial dysfunction both in vivo and in vitro [30]. The development of inflammation appears to be a mechanism underlying the pathophysiology of CVD [35]. Therefore, decreasing serum TFAs may be an approach for modifying inflammatory response and associated disorders such as CVD and DM [16].

*trans*-Linoleic acid (C18:2n6t) is directly related to plasminogen activating inhibitor-1 (PAI-1) activity [33]. PAI-1 is produced in the liver and in adipose tissue and plays a crucial role in preventing fibrin clot breakdown, thereby supporting thrombus formation [33]. It has been confirmed in mice that high consumption of TFAs (elaidic acid) stimulates thrombus formation in the carotid artery compared to* cis*-fatty acid diet [33]. Industrially produced* trans*-fatty acids may induce endothelial dysfunction as assessed by flow-mediated vasodilatation and the upregulation of proinflammatory molecules production [36]; hence, the activation of proinflammatory cytokines implicates the link between vascular inflammation, atherosclerosis development, and thrombosis process, including rise in PAI-1 expression [33, 36].

Average* trans*-fat intake varies worldwide, with some of the highest intake reported in Egypt, followed by Pakistan, Canada, Mexico, and Bahrain. Several island nations in the Caribbean including Barbados and Haiti have lower consumption, followed by East Sub-Saharan African nations such as Ethiopia and Eritrea [37]. Commercial foods are a major source of* trans*-fat in high-income countries, while intakes in low- and middle-income counties are principally derived from home and street vendors' use of inexpensive partially hydrogenated cooking fats [38, 39].

Increased levels of* trans*-palmitoleic acid (16:1n-7* trans*) derived from dairy fat have been associated with lesser risk of type 2 diabetes [9]. The prospective Cardiovascular Health Study [40] reported that plasma phospholipid* trans*-palmitoleic acid was related inversely with insulin resistance. In the Multi-Ethnic Study of Atherosclerosis [41],* trans*-palmitoleic acid was related with less incident diabetes and inversely with plasma fasting insulin. 16:1n-7* trans* (*trans*-palmitoleic acid) and 18:1n-7 (vaccenic acid) levels have been directly correlated with the number of full-fat dairy servings in one investigation [42], while another investigation found no significant change in plasma* trans*-fatty acids and fatty acid levels in general with increased dairy food intake [43]. Plasma phospholipid elaidic acid concentrations, the main TFA isomer occurring during partial hydrogenation of vegetable oils found in a myriad of industrial foods, were positively associated with the intake of highly processed foods within the European Prospective Investigation into Cancer and Nutrition (EPIC) cohort [44, 45].

Our study has several strengths. First, we used serum measurements of TFAs concentration as a marker of intake, a preferred measure of intake over questionnaires because of objectivity and absence of recall bias [46]. Our study is based on a nationally representative survey with large sample size. The study is sufficiently powered to test the associations. The selection of the participants was based on random sampling of the general population and therefore the results obtained from nationally representative samples can be extrapolated to the general population. Potential limitations include the cross-sectional design which does not allow inference about causality. We did not have repeated measures of TFAs in the same subjects after several follow-up years to elucidate temporality of these findings.

## 5. Conclusion

The correlation between objectively measured TFAs levels and markers of inflammation in the current study supports the hypothesis that TFAs may contribute to common chronic diseases by exacerbating the underlying chronic inflammatory processes. Control of TFAs intake may therefore have a role in the prevention of chronic disease via action on chronic inflammation. In this regard, action should target all exogenous sources of TFAs, either naturally occurring dairy or industrially processed.

## Figures and Tables

**Table 1:** Age- and sex-adjusted mean of serum *trans*-fatty acids across quartiles of hs-CRP and fibrinogen.

Variables	Quarters of hs-CRP					Quarters of fibrinogen				
	Q1	Q2	Q3	Q4	*p*	Q1	Q2	Q3	Q4	*p*
Mean ± SD	0.049 ± 0.014	0.16 ± 0.044	0.37 ± 0.088	1.4 ± 0.95		280.3 ± 29.2	340.1 ± 13.2	386.1 ± 14.8	478.2 ± 63.9	
*trans*-9-hexadecenoic acid	1.79 ± .025	1.91 ± .023	1.95 ± 0.003	1.91 ± 0.002	<0.001	1.85 ± .030	1.95 ± .029	1.93 ± 0.005	1.92 ± 0.006	<0.001
*trans*-11-octadecenoic acid	3.56 ± .026	3.67 ± 0.010	3.64 ± 0.008	3.57 ± 0.007	<0.001	3.60 ± .030	3.67 ± .033	3.62 ± 0.001	3.60 ± 0.011	<0.001
*trans*-9-octadecenoic acid	3.39 ± .027	3.52 ± 0.001	3.51 ± 0.004	3.49 ± 0.001	<0.001	3.47 ± .034	3.56 ± .032	3.54 ± .030	3.48 ± 0.001	<0.001
*trans*-9-, *trans*-12-octadecadienoic acid	.879 ± .025	1.01 ± .023	1.019 ± .022	1.023 ± .024	<0.001	1.048 ± .030	1.051 ± .029	1.038 ± 0.008	.952 ± 0.001	<0.001

*p* values for linear trend across quarters of hs-CRP. Variables were compared across quarters of hs-CRP and fibrinogen using analysis of covariance (ANCOVA) test.

## Abbreviations

ANCOVA: Analysis of covariance
CVD: Cardiovascular disease
DM: Diabetes mellitus
Hs-CRP: high sensitivity C-reactive protein
IL-6: Interleukin-6
NCHS: National Center for Health Statistics
NF-*κ*B: Nuclear factor-*κ*B
NHANES: National Health and Nutrition Examination Survey
PAI: Plasminogen activating inhibitor
PFB-Br: Pentafluorobenzyl bromide
TFAs: * trans*-Fatty acids.

## Data Access

NHANES data are publicly available already.
